# Brain 3-Mercaptopyruvate Sulfurtransferase (3MST): Cellular Localization and Downregulation after Acute Stroke

**DOI:** 10.1371/journal.pone.0067322

**Published:** 2013-06-21

**Authors:** Heng Zhao, Su-Jing Chan, Yee-Kong Ng, Peter T.-H. Wong

**Affiliations:** 1 Department of Pharmacology, Yong Loo Lin School of Medicine, National University Health System, National University of Singapore,Singapore, Singapore; 2 Department of Anatomy, Yong Loo Lin School of Medicine, National University Health System, National University of Singapore,Singapore, Singapore; Massachusetts General Hospital/Harvard Medical School, United States of America

## Abstract

3-Mercaptopyruvate sulfurtransferase (3MST) is an important enzyme for the synthesis of hydrogen sulfide (H_2_S) in the brain. We present here data that indicate an exclusively localization of 3MST in astrocytes. Regional distribution of 3MST activities is even and unremarkable. Following permanent middle cerebral artery occlusion (pMCAO), 3MST was down-regulated in both the cortex and striatum, but not in the corpus collosum. It appears that the down-regulation of astrocytic 3MST persisted in the presence of astrocytic proliferation due to gliosis. Our observations indicate that 3MST is probably not responsible for the increased production of H_2_S following pMCAO. Therefore, cystathionine β-synthase (CBS), the alternative H_2_S producing enzyme in the CNS, remains as a more likely potential therapeutic target than 3MST in the treatment of acute stroke through inhibition of H_2_S production.

## Introduction

Ischemic stroke occurs when the blood supply to a particular area of the brain stops due to occlusion of a blood vessel. It has been reported that poor clinical outcome in acute stroke patients is strongly associated with high plasma homocysteine (Hcy) and cysteine (Cys) levels [1–3]. In animal studies, the administration of cysteine increased the infarct volume after experimental stroke induced by permanent middle cerebral artery occlusion (pMCAO), which could be attenuated by aminooxyacetic acid, an inhibitor of the enzyme cystathionine β-synthase (CBS). As CBS can produce hydrogen sulfide (H_2_S) using Cys and/or Hcy as substrates [4–6], these observations indicate that the Cys effect may be due to its conversion to H_2_S [3]. Moreover, administration of NaHS, an H_2_S donor, instead of Cys, similarly increased infarct volume after pMCAO [7]. H_2_S, although well-known to be a toxic gas, is now recognized to be present in mammalian tissues and has important physiological functions especially in the cardiovascular system and the central nervous system (CNS) [8,9]. It is an important neuromodulator which facilitates the induction of hippocampal long-term potentiation (LTP) by enhancing the activity of NMDA receptors in neurons and promotes the influx of Ca^2+^ into astrocytes (calcium wave) [4,10]. It is known that H_2_S may be produced by the action of 2 key enzymes in the brain, namely, the pyridoxal-5'-phosphate (PLP)-dependent CBS [4,11], and the PLP-independent 3-mercaptopyruvate sulfurtransferase (3MST). 3-Mercaptopyruvate (3-MP) is converted from cysteine by the action of cysteine aminotransferase (CAT) [12]. It has been reported that H_2_S produced by 3MST may be readily stored as bound sulfane sulfur, which in turn can rapidly release H_2_S on stimulation. Thus, cells expressing 3MST and CAT have an increased level of bound sulfane sulfur [12]. However, it is not known what changes occur in 3MST expression under ischemic conditions in the brain. As H_2_S is known to increase after stroke [7], we hypothesized that the expression of 3MST might increase if 3MST is the major source of H_2_S under such conditions. In this article, we report the regional distribution of 3MST activities and the cellular localization of 3MST, and its expression in the striatum and cortex before and after pMCAO.

## Methods

### Ethics Statement

All animal experimental procedures in this study were approved by the Institutional Animal Care and Use Committee of the National University of Singapore.

### 3MST assay

3MST activities in tissue homogenates were measured according to Westrop et al. [13] with significant modifications as follow. All incubations were performed in reaction tubes fitted with air-tight serum caps and plastic center wells. The centre well contained a folded 2cm x 2.5cm filter paper (Whatman No. 1) wetted with 0.5ml of 1% (w/v) zinc acetate in 12% NaOH for trapping evolved H_2_S. Brain homogenate (300µl, 14.3% w/v) in 50mM potassium phosphate buffer (pH6.8) was mixed with 3-MP (2mM, sodium salt, Sigma-Aldrich) and 2-mercaptoethnol (10mM, Sigma-Aldrich) with or without 2-ketobutyric acid (40 mM), an uncompetitive inhibitor of 3MST [14,15], in the reaction tube in an ice bath. Total volume was 0.4 ml. The reaction tube was then flushed with N_2_ for 20s and then capped. The reaction was initiated by transferring the tube to a shaking bath at 37°C. After incubating for 90 min, the reaction was stopped by injecting trichloroacetic acid (0.5 ml, 50% w/v) through the serum cap. After 1 h incubation at 37°C to allow complete trapping of H_2_S, the centre well was taken out. N,N-Dimethyl-p-phenylenediamine sulphate in 7.2M HCl (0.5 ml, 20mM) and FeCl_3_ in 1.2M HCl (0.5ml, 30mM) were added and left in the dark at room temperature for 20min. Finally the absorbance at 670nm was determined with a spectrophotometer (Epoch, BioTek). Blanks were obtained by replacing brain homogenate with buffer. Calibration curve was obtained using NaHS (0-1mM).

### pMCAO

Male Sprague Dawley rats (250-280 g) were randomly assigned into pMCAO group or sham control group. A subtemporal approach was used to induce the permanent occlusion of the left middle cerebral artery (MCA) [7,16]. The rats were anaesthetized with ketamine (75 mg/kg i.p.) and xylazine (10 mg/kg i.p.). A craniectomy was extended dorsally up to the first major branch of the MCA. Then the dura was opened with a bent 26-gauge needle and the arachnoid membrane was carefully removed. The MCA was cauterized with an electrocauterizer without damaging the brain surface and then cut. The site of the occlusion is between the inferior cerebral vein and olfactory tract. The sham group was operated in the same way as the experimental groups but with the MCA left intact.

### Immunohistochemistry

The localizations of 3MST were examined in rat brain sections using immunohistochemistry. Brain were removed from pMCAO or sham-operated rats transcardially perfused with ringer solution and 2% paraformaldehyde and immersion-ﬁxed in 2% paraformaldehyde for 4 h, dehydrated in 15% sucrose overnight, embedded in OCT compound (Sakura Finetek, Torrance, CA, USA), cryosectioned to 30µm-thick sections, and thaw mounted on gelatin-coated slides. Non-specific binding was blocked by incubating the section in 5% goat serum for 1h. This was followed by overnight incubation with 3MST antibody (1:200, Sigma-Aldrich) and one of the following three antibodies: anti-NeuN (1:200, Millipore, MAB377), a postmitotic neuronal nuclei marker; anti-glial ﬁbrillary acidic protein (GFAP, 1:200, Sigma-Aldrich), a speciﬁc type of fibrillary protein used as astrocyte marker; and anti-OX-42 (CD11b, 1:200, AbD Serotec), a monoclonal antibody recognizing the C3bi complement receptor expressed by microglial cells. This was followed by incubation of FITC conjugated goat anti-rabbit IgG used against 3MST antibody and Cy3 conjugated goat anti-mouse IgG used against anti-NeuN, anti-GFAP or anti-OX 42 for 1h for ﬂuorescent labelling. Sections were then incubated in 4’,6-Diamidino-2-phenylindole dihydrochloride (DAPI, Sigma-Aldrich, 0.5 µg/ml in PBS) for 5 minutes, mounted and observed under a confocal microscope (Olympus, Tokyo, Japan).

### Western blot analysis

Brain tissues (cortex or striatum) were homogenized in cold lysis buffer [10 mM HEPES, pH 7.9 with 1.5 mM MgCl_2_ and 10 mM KCl, containing protease inhibitor cocktails (Roche Diagnostics GmbH, Mannheim, Germany)]. The lysates were centrifuged for 10 min at 14000×g. The cytoplasmic fractions containing equal amounts of protein, as determined by the Nano-Drop method (Thermo Scientific), were separated by 10% SDS/PAGE and transferred onto a nitrocellulose membrane (Amersham Biosciences, Buckinghamshire, UK). After incubating in 10% milk at room temperature in TBST buffer (10 mM Tris-HCl, 120 mM NaCl, 0.1% Tween-20, pH 7.4) for 1 h, the membranes were incubated with antibodies against 3MST (Sigma-Aldrich) at 4°C overnight, and then incubated with 1:1000 dilutions of HRP-conjugated anti-rabbit IgG at room temperature for 1h. The visualization was detected using a high quality fluorescent camera (UVItec Cambridge). The density of the bands on the membranes was quantified by densitometry analysis of the scanned blots using UVIband software. All protein levels were normalized to the corresponding β-actin bands (Sigma-Aldrich). For Western blotting, the contralateral side of the pMCAO brain was used as control instead of sham animals in order to reduce the number of animals used to a minimum as preliminary data showed no significant difference between the contralateral side and sham-control.

### Statistical analysis

All statistical analyses were performed by using Excel 2013 (Microsoft, Redmond, WA) and SPSS 20 (IBM, Armonk, NY). Differences between two groups were analyzed with Student’s t-test. Differences between three or more groups were analyzed with one-way analysis of variance (ANOVA). Post hoc multiple comparisons were made by using the Bonferroni test.

## Results

**Figure 1:**
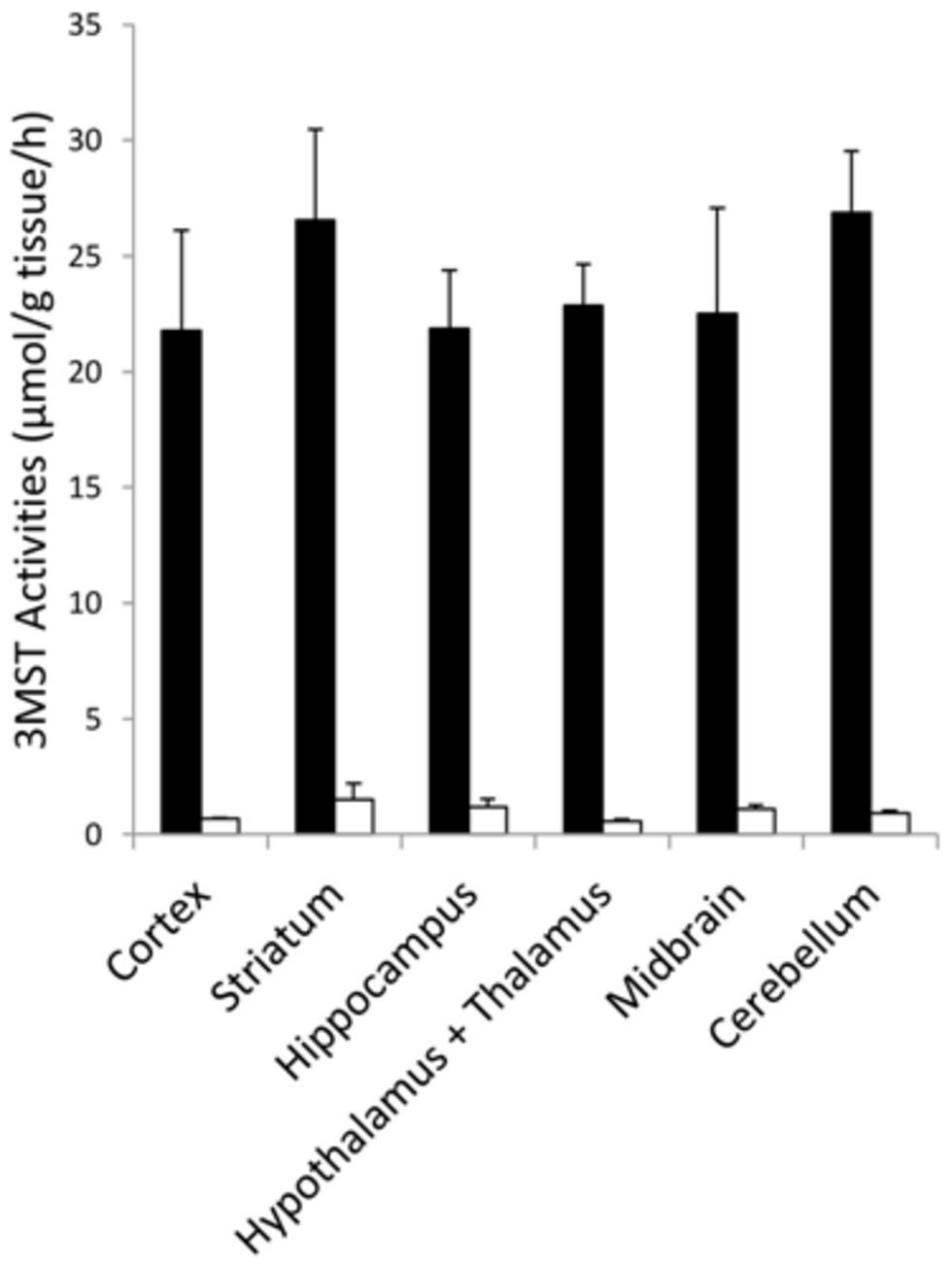
3MST activities in six different regions of the rat brain. 3MST activities were measured biochemically in tissue homogenates in the absence (solid bars) and presence of 2-ketobutyric acid (open bars) as described in Methods; N=3.

3MST activities were detected in all six brain regions examined as shown in Figure 1. There appeared to be no significant differential distribution with all regions exhibiting mean activities in the range of about 21 to 26 µmol/g tissue/h (Figure 1). In the presence of 2-ketobutyric acid, 3MST activities were almost completely inhibited showing that no other enzymes contributed to the production of H_2_S under the assay conditions used.

3MST activities were measured biochemically in tissue homogenates in the absence (solid bars) and presence of 2-ketobutyric acid (open bars) as described in Methods; N=3.

**Figure 2:**
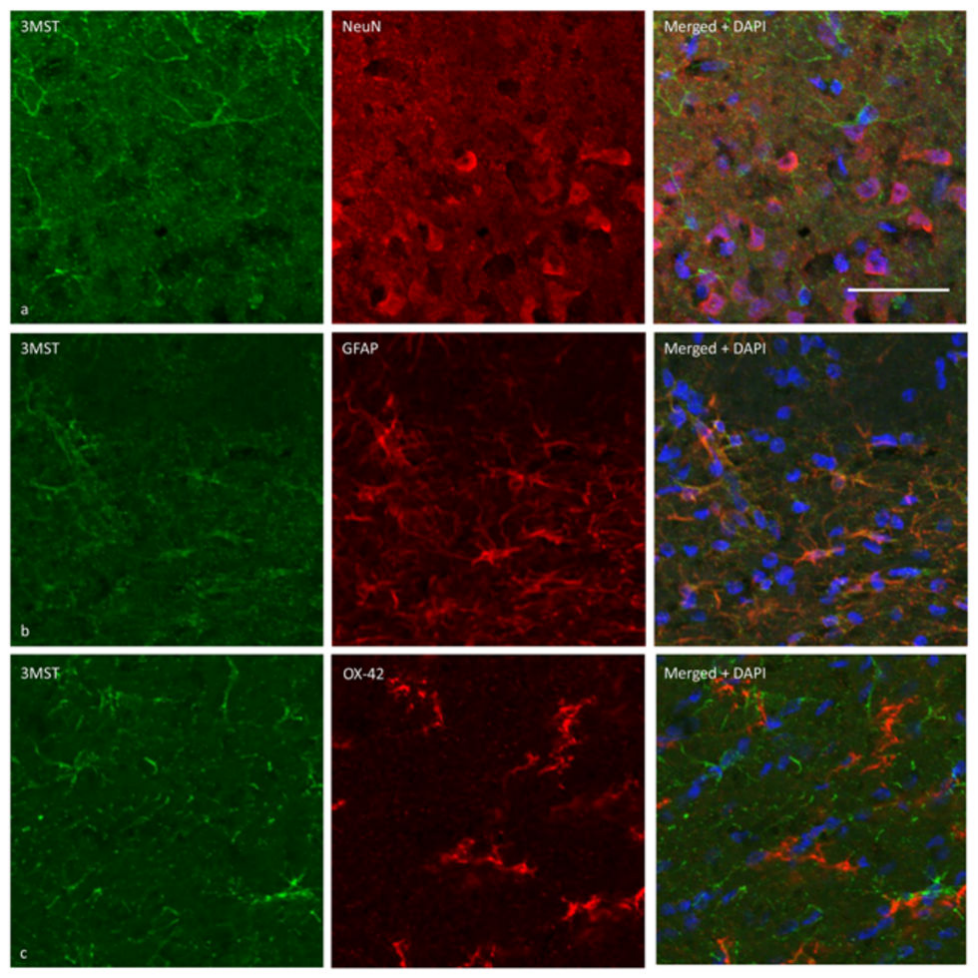
Cellular localization of 3MST using dual-fluorescent immunohistochemical labelling on rat cortical sections. (a) 3MST immunoreactivity (green) does not colocalize with NeuN-immunoreactive neurons (red). (b) 3MST immunoreactivity colocalizes with GFAP-immunoreactive astrocytes (red). (c) 3MST does not colocalize with OX42-immunoreactive mciroglial cells (red). Scale bar = 50µm. All sections were observed with an Olympus confocal fluorescent microscope.

**Figure 3:**
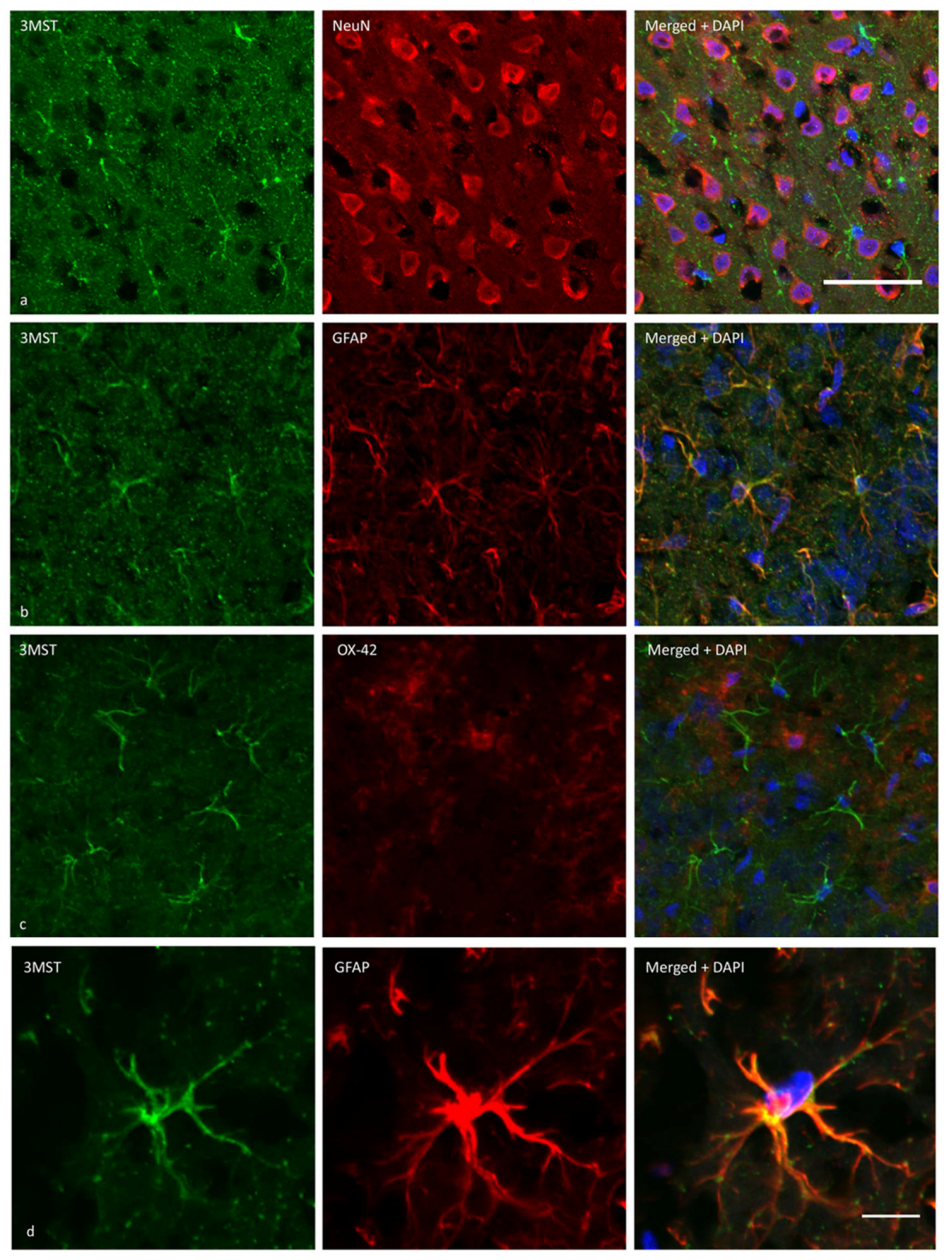
Cellular localization of 3MST using dual-fluorescent immunohistochemical labelling on rat striatal sections. (a) 3MST immunoreactivity (green) does not colocalize with NeuN-immunoreactive astrocytes (red). (b) 3MST immunoreactivity (green) colocalizes with GFAP-immunoreactive neurons (red). (c) 3MST (green) does not colocalize with OX42-immunoreactive mciroglial cells (red). Scale bar = 50µm. All sections were observed with an Olympus confocal fluorescent microscope. (d) High magnification photomicrograph showing an astrocyte stained positive for both 3MST and GFAP. Scale bar = 10 µm.

The expression of 3MST in brain cells was demonstrated using immunohistochemical staining. Figures 2 and 3 show 3MST immunoreactivity in the cortex and striatum, respectively. By double immunostaining, 3MST was demonstrated to localize in astrocytes as it colocalized with GFAP immunoreactivities. In contrast, 3MST immunoreactivity did not colocalize with NeuN or OX42, indicating that it was not expressed in neurons or microglia. At the subcellular level, 3MST immunoreactivity appears to be cytoplasmic and is present in both the processes and cell soma of the astrocytes.

**Figure 4:**
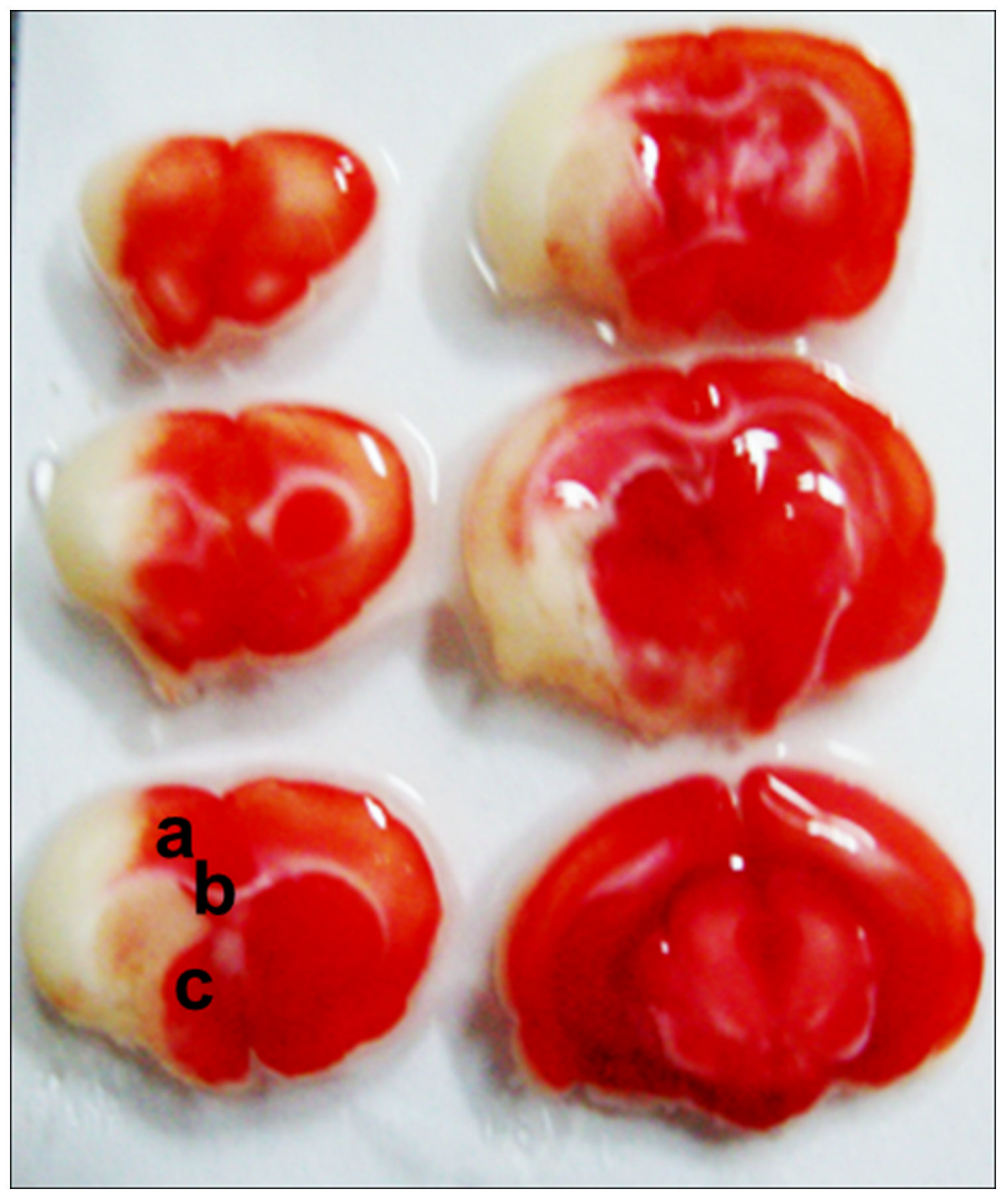
A TTC-stained sections (2 mm) of a rat forebrain after pMCAO. The infracted regions are not stained by TTC thus appear white. Photomicrographs presented in Figs. 5 – 7 are taken from the peri-infarct areas at the level of +1mm from Bregma as indicated. a: Cortex; b: corpus callosum; c: striatum.

**Figure 5:**
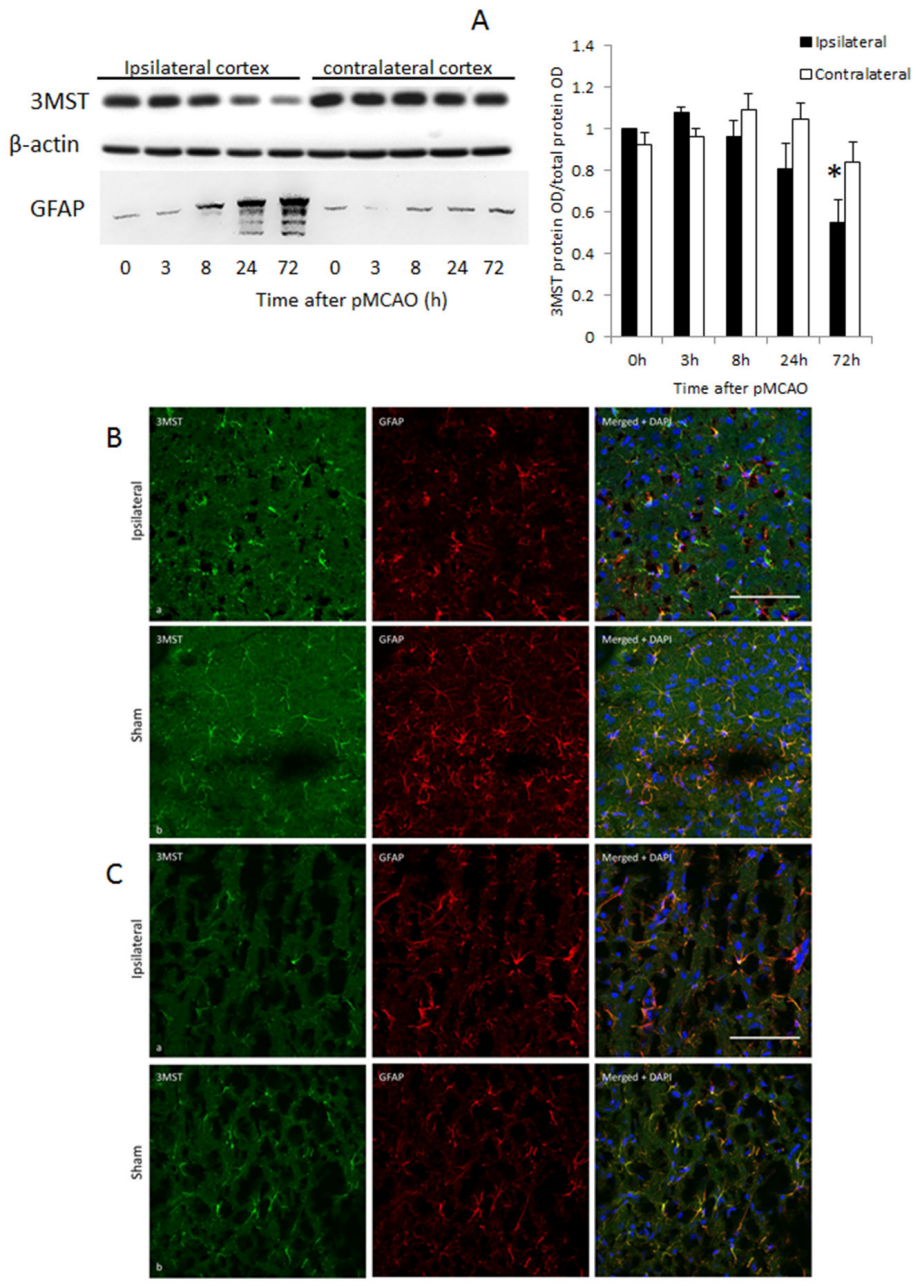
3MST expression in the ipsilateral cortex at various time points after pMCAO. (A) Representative western blot bands are shown in the top panel. N=4. *p<0.05 against the contralateral side. (B) 3MST immunoreactivity (green) in the ipsilateral cortex 24 h after pMCAO (top row) and sham-operated (bottom row) cortex showing co-localization with GFAP-immunoreactive astrocytes (red). Scale bar = 100µm. (C) 3MST immunoreactivity (green) in the ipsilateral cortex 72 h after pMCAO (top row) and sham-operated (bottom row) cortex showing co-localization with GFAP-immunoreactive astrocytes (red). Scale bar = 100µm.

**Figure 6:**
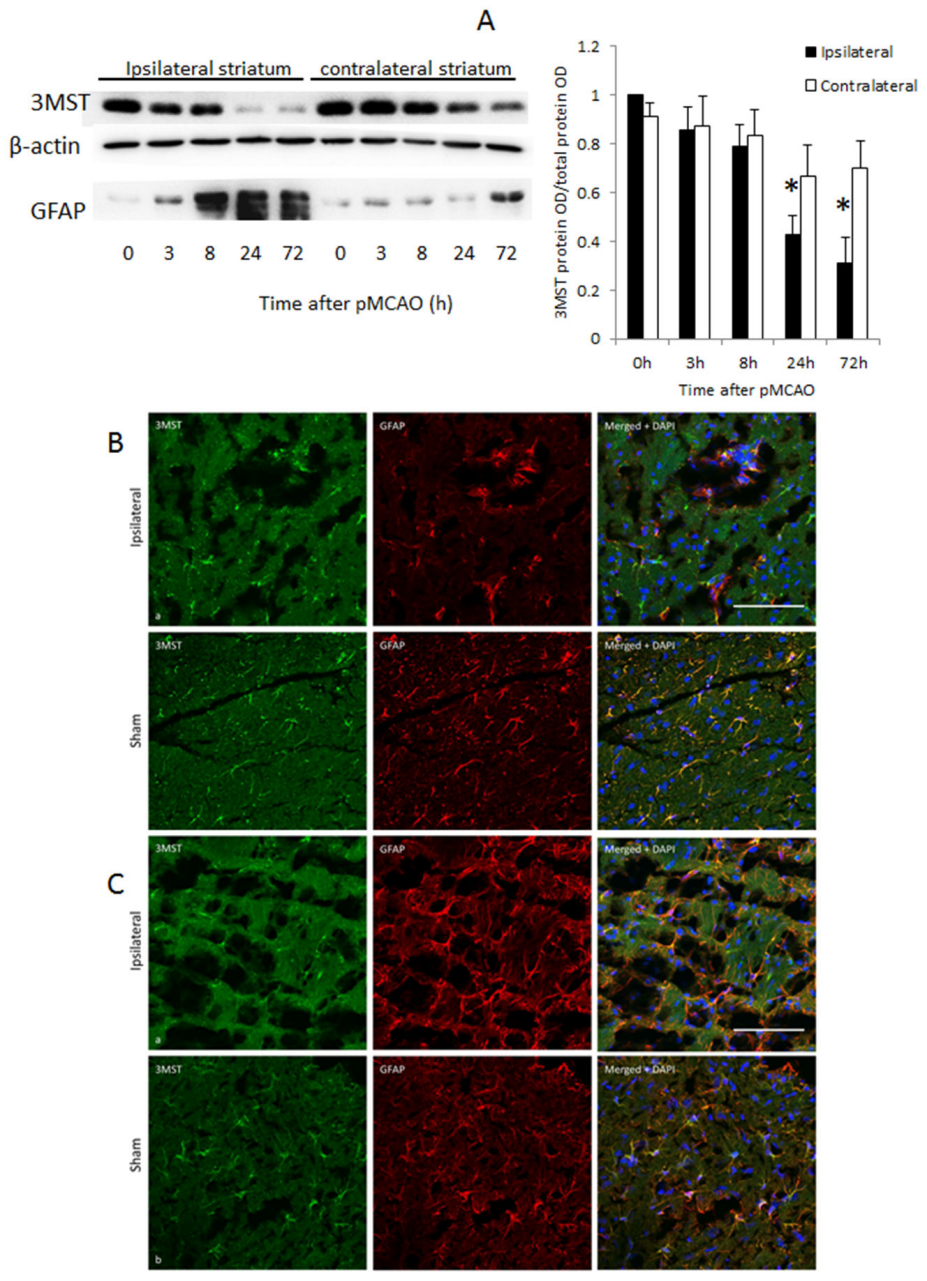
3MST expression in the ipsilateral striatum at various time points after pMCAO. (A) Representative western blot bands are shown in the top panel. N=4. * p<0.05 against the contralateral side. (B) 3MST immunoreactivity (green) and GFAP-immunoreactive astrocytes (red) in the ipsilateral striatum 24 h after pMCAO (a) and sham-operated striatum (b). Merged photomicrographs (left) show co-localization of 3MST and GFAP immunoreactivity (yellow). Scale bar = 100µm. (C) 3MST immunoreactivity (green) and GFAP-immunoreactive astrocytes (red) in the ipsilateral striatum 72 h after pMCAO (a) and sham-operated striatum (b). Merged photomicrographs (left) show co-localization of 3MST and GFAP immunoreactivity (yellow). Scale bar = 100µm.

**Figure 7:**
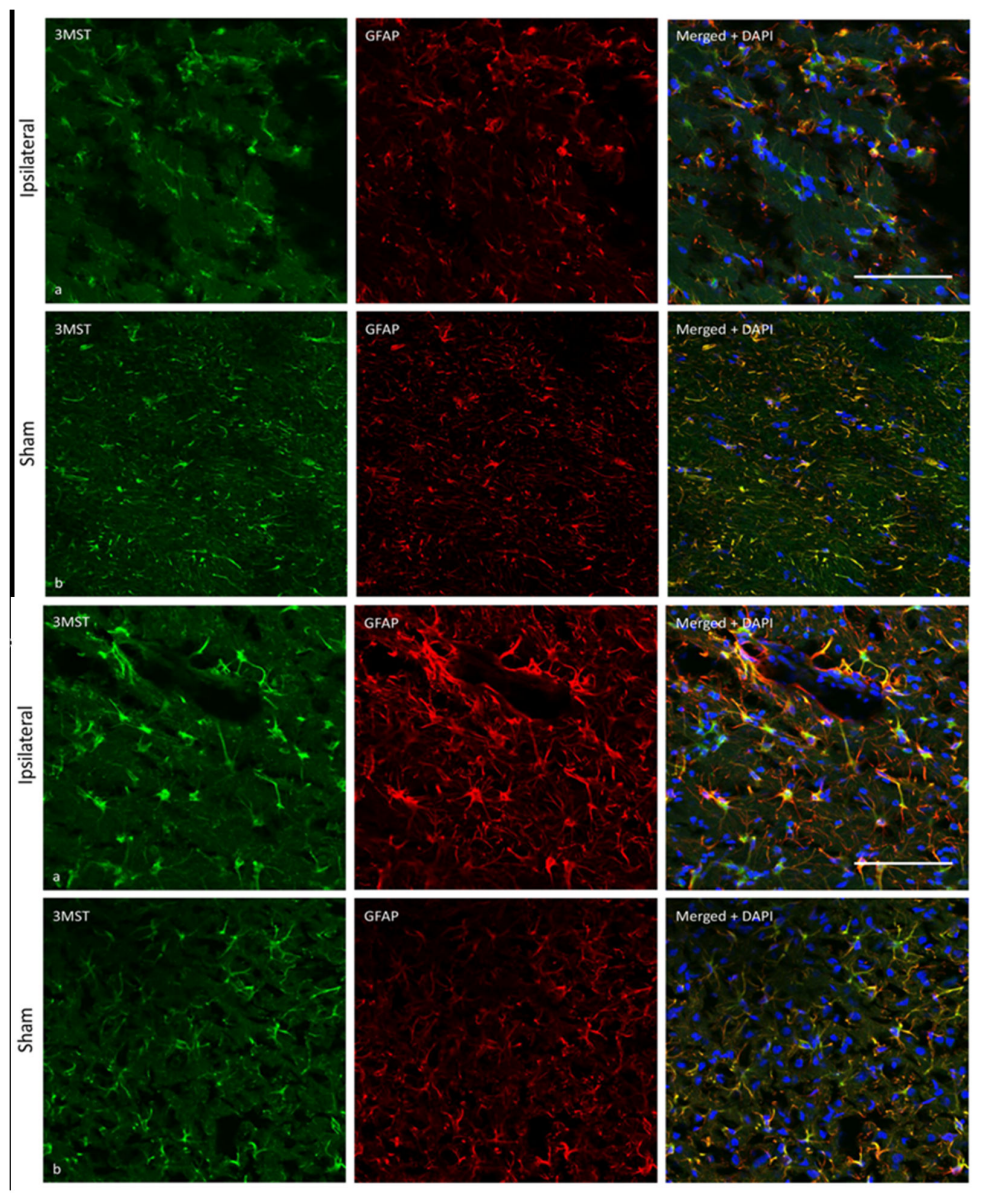
3MST expression in the corpus callosum. (A) 3MST immunoreactivity (green) and GFAP-immunoreactive astrocytes (red) in the ipsilateral corpus callosum 24 h after pMCAO (a) and sham-operated corpus callosum (b). Merged photomicrographs (left) show co-localization of 3MST and GFAP immunoreactivity (yellow). Scale bar = 100µm. (B) 3MST immunoreactivity (green) and GFAP-immunoreactive astrocytes (red) in the ipsilateral corpus callosum 72 h after pMCAO (a) and sham-operated corpus callosum (b). Merged photomicrographs (left) show co-localization of 3MST and GFAP immunoreactivity (yellow). Scale bar = 100µm.

Consistent with previously findings, when rats were subjected to pMCAO, the infracted areas involved the cortex, striatum, corpus callosum and hippocampus [7] as shown in Figure 4. When the expression of 3MST was investigated in the cortex and striatum after pMCAO by Western blot analysis, it was found that 3MST was significantly down-regulated by about 40-50% at 72h in the cortex and at 24 h in the striatum. In contrast, expression of GFAP increased progressively from 8 h onward reaching very high levels at 24 and 72 h after pMCAO (Figures 5A and 6A). While the western blot analysis was performed in whole cortex and striatum, immunohistochemical staining was studied in the peri-infarct areas as indicated in Figure 4. Immunostaining results are generally consistent with the Western blot analysis and they clearly show that 3MST immunoreactivity remained colocalized with astrocytes under ischemic conditions as shown in Figures 5–7).

The infracted regions are not stained by TTC thus appear white. Photomicrographs presented in Figures 5–7 are taken from the peri-infarct areas at the level of +1mm from Bregma as indicated. a: Cortex; b: corpus callosum; c: striatum.

At 24 h after pMCAO, it was observed that both 3MST and GFAP immunoreactivities were much reduced in both the cortex and striatum (Figures 5B and 6B) despite the increase in GFAP expression observed in Western blot analysis. This suggests that at this time point, astrocyte proliferation was still confined to the core infarct areas but not in the peri-infarct areas. At 72 h post-pMCAO, GFAP immunoreactivity was quite comparable to control, reflecting a significant level of astrocyte proliferation in the peri-infarct areas. However, 3MST immunoreactivity remained low at this time point indicating that 3MST expression continued to be suppressed despite astrocyte proliferation.

In contrast to the cortex and striatum, immunostaining results show that 3MST expression did not appear to be affected in the corpus callosum at both 24 and 72 h after pMCAO (Figure 7). Therefore, it appears that the suppression of 3MST expression is not a general phenomenon occurring in all peri-infarct regions following pMCAO. However, the possibility of a change in 3MST expression in this region at a later time point may not be ruled out.

## Discussion

It has been known that H_2_S may be synthesized through the actions of 3 enzymes, CBS, 3MST and cystathionine γ-lyase (CSE) [12,17]. CSE, while being important in the cardiovascular system [8], is considered a minor contributor to H_2_S synthesis in the brain based on its low level of expression [4]. CBS can produce H_2_S either by hydrolyzing its substrate Cys or by condensation of Cys and Hcy. Kinetic studies have shown that the latter appeared more efficient [18]. On the other hand, 3MST produce H_2_S from 3-MP with pyruvate as the by-product. 3-MP is produced by CAT from Cys. Therefore, the two pathways can be commonly regulated by Cys availability. However, the regulation of H_2_S synthesis in the brain is not well understood and has been reported to be closely associated with intracellular level of Ca^2+^ [19].

Our finding of astrocytic expression of 3MST contradicts an earlier report that 3MST is localized to neurons in many areas of the mouse brain and spinal cord [12]. In the cortex, 3MST immunoreactivity was reportedly present in pyramidal neurons in layers II/III and V, and in layers I-VI of the neocortical areas [12]. Although colocalization with NeuN was not performed in the previous study [12], the immunohistochemical staining presented are convincing. It is difficult to know the cause of such discrepancy and any explanation can only be speculative. However, an astrocytic localization of 3MST seems consistent with the even and unmarkable regional variation of the in vitro 3MST activities (Figure 1).

We have previously reported that H_2_S level in cortical tissues was increased by 2-fold 24h after pMCAO. This increase was not associated with an upregulation of CBS expression [7]. Our present observation that 3MST is significantly downregulated under the same conditions would indicate that 3MST is not likely to be responsible for the increased production of H_2_S under ischemic conditions. This is interesting as CBS and 3MST are supposedly the predominant enzymes for H_2_S synthesis. However, we have now obtained preliminary data that show an upregulation of the truncated CBS (45kDa) (unpublished) while full-length CBS (62kDa) remained unchanged as reported previously [7]. It has been reported that the truncation of CBS was associated with increased CBS activities [20]. Most recently, Perna et al. [21] reported that NaHS down-regulated both 3MST and CSE in cultured endothelial cells. Therefore, it is a distinct possibility that the observed down-regulation of 3MST is caused by a high level of H_2_S produced through CBS truncation and activation.

In conclusion, 3MST appears not to be instrumental in the acute increase in H_2_S production in the ischemic brain. As inhibition of H_2_S production may lead to a reduction in ischemic damage, CBS remains as a more likely potential therapeutic target than 3MST in the treatment of acute stroke.
